# Tin–zinc-oxide nanocomposites (SZO) as promising electron transport layers for efficient and stable perovskite solar cells†

**DOI:** 10.1039/c9na00182d

**Published:** 2019-05-22

**Authors:** Ahmed E. Shalan, Ayat N. El-Shazly, Mohamed M. Rashad, Nageh K. Allam

**Affiliations:** Central Metallurgical Research and Development Institute (CMRDI) P. O. Box 87, 11422, Helwan Cairo Egypt a.shalan@cmrdi.sci.eg; Energy Materials Laboratory, School of Sciences and Engineering, The American University in Cairo (AUC) 11835 New Cairo Egypt nageh.allam@aucegypt.edu

## Abstract

Tin–zinc-oxide nanocomposites (SZO) with various Sn : Zn ratios were successfully fabricated and tested as electron transport layers (ETLs) in perovskite solar cells (PVSCs). The fabricated nanocomposites showed good crystallinity, good contact between layers, good electrical conductivity, and favorable light absorption, resulting in an enhancement in the net efficiency of CH_3_NH_3_PbI_3_ (MAPI)-based perovskite solar cells. The device made of SZO–Sn_0.05_ as an ETL showed a maximum power conversion efficiency (PCE) of 17.81% with a short-circuit current density (*J*_sc_) of 23.59 mA cm^−2^, an open-circuit voltage (*V*_oc_) of 1 V, and a fill factor (FF) of 0.754. However, the ETL containing lower Sn ratios showed PCEs of 12.02, 13.80 and 15.86% for pure ZnO, SZO–Sn_0.2_ and SZO–Sn_0.1_, respectively. Meanwhile, the reproducibility of 30 fabricated devices proved the outstanding long-term stability of the cells based on SZO nanocomposites, retaining ≈85% of their PCE over 1200 h of operation. In addition, the incident-photon-to-current efficiency (IPCE) exceeded 90% over the entire wavelength range from 400 to 800 nm. The enhancement in the PCE of the fabricated PVSCs can be ascribed to the large surface area of the SZO nanoparticles, high charge extraction efficiency, and suppression of charge recombination provided by SnO_*x*_. The current results suggest that our synthesized tin–zinc-oxide nanocomposite is an effective electron transport layer for efficient and stable perovskite solar cells.

## Abstract 2

Tin–zinc-oxide nanocomposites (SZO) with various Sn : Zn ratios were successfully fabricated and tested as electron transport layers (ETLs) in perovskite solar cells (PVSCs).

## Graphical abstract

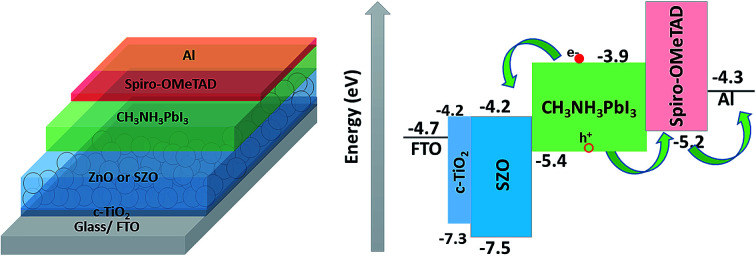

## Introduction

Organic–inorganic hybrid halide perovskite solar cells (PVSCs) fabricated using CH_3_NH_3_PbX_3_ (X = Cl, Br, I) have attracted great attention due to their low fabrication cost, achieving a certified power conversion efficiency (PCE) of 22.1%.^1^ The high performance of PVSCs is related to the exceptional properties of the used perovskite material, being an excellent absorber and having a direct band gap, high absorption coefficient, and excellent carrier transport.^2^ However, the long-term stability under realistic operating conditions is a great challenge that needs to be addressed.^3^ A number of architectures have been proposed to assemble PVSCs. The widely used device assembly consists of an electron transport layer (ETL), a perovskite layer as the light harvesting material, and a hole transport layer (HTL). Controlling the quality of the perovskite/ETL interface is a key point to achieve long-term stability.^4^ Also, the dual functionality of the ETL, as either a hole blocking or an electron transfer layer, enhances the fill factor and the generated photocurrent by suppressing the recombination rate of charge carriers.^5^ To this end, various materials have been investigated as ETLs such as TiO_2_, ZnO, SnO_2_, Nb_2_O, and WO_3_.^6–10^ Most PVSCs utilize TiO_2_ as a scaffold (ETL), which was reported to have a low electron mobility of 20 cm^2^ V^−1^ s^−1^ and requires high sintering temperature (usually above 450 °C).^11,12^ Notably, ZnO has attracted considerable interest due to its good transparency, high electron mobility (200–300 cm^2^ V^−1^ s^−1^), and strong room temperature luminescence, and can be made crystalline under mild conditions.^13,14^ However, the efficiency of the PVSCs based on ZnO as an ETL is still relatively low due to the chemical instability of ZnO in acidic medium, which causes decomposition of methyl ammonium ions.^15–18^ On the other hand, SnO_*x*_ is a wide band gap semiconductor with a more positive conduction band (CB) edge, making it a better option as an ETL than ZnO and TiO_2_.^19^ SnO_*x*_ was shown to be more efficient in collecting electrons in electrochemical cells with high durability in an ambient environment.^19^ However, upon the use of SnO_*x*_, the assembled cells showed low PCE, which can be ascribed to the inherent low conduction band edge, resulting in a fast recombination process and low open-circuit voltage.^20^ Therefore, the combination of the two semiconductors (ZnO and SnO_*x*_) into a composite material is expected to overcome the above mentioned limitations. The use of SnO_*x*_–ZnO (SZO) nanocomposites was shown to result in a higher photocatalytic activity than that of the individual ZnO and SnO_*x*_ counterparts, through reducing the electron–hole recombination rate.^21–23^ In addition, SZO nanocomposites were shown to be promising candidates as anode materials in rechargeable lithium ion batteries.^24,25^ Moreover, various SZO heterostructures have successfully been applied in both liquid and solid state dye-sensitized solar cells to enhance the photo-conversion efficiency of the fabricated devices.^26–30^ Similarly, the use of SZO as an ETL in PVSCs is an interesting subject to be investigated.^31^ To this end, and to the best of our knowledge, there is only one report in the literature on the use of a ZnO/SnO_2_ nanocomposite.^31^ In that report, SnO_*x*_ was added to the ZnO structure as a photoanode in PVSCs with a PCE of 14%.^31^ However, this study was limited to only one composition with no variation in the Zn : Sn ratio, making it very hard to tell whether the improved performance was due to the cell assembly or the composition of the ZnO/SnO_2_ nanocomposite, which necessitates further investigation. In this regard, the study of tin–zinc-oxide (SZO) nanocomposites with different Sn contents as an ETL for PVSCs is very essential to identify the best composition that enables the effective utilization of long-wavelength photons. Herein, we synthesized SZO nanocomposites with different Sn contents *via* a simple and cost-effective co-precipitation wet chemical method. The synthesized SZO nanocomposites were tested as ETLs in n–i–p PVSC devices. The synthesized nanocomposites and the assembled devices were extensively characterized and compared to the reports in the literature.^31^

## Experimental section

### Chemicals and materials

Zinc sulphate heptahydrate (ZnSO_4_·7H_2_O) and stannous chloride dihydrate (SnCl_2_·2H_2_O) were used as received from Dop Organic Chemical as a source of Zn^2+^ and Sn^2+^ ions, respectively, during the co-precipitation pathway. As a precipitating agent, ammonium hydrogen carbonate (NH_4_HCO_3_) (99%, Riedel-de Haën) was incorporated in the preparation reaction. The perovskite (CH_3_NH_3_PbI_3_, MAPI) solution was prepared by adding CH_3_NH_3_I, which was synthesized in the lab, to PbI_2_ (99%, Aldrich) in an equimolar percentage in dry *N*,*N*-dimethyl formamide (DMF, Aldrich) as described elsewhere in our previous work.^32–34^ In addition, spiro-OMeTAD (2,2′,7,7′-tetrakis-(*N*,*N*-di-4-methoxyphenylamino)-9,90-spirobifluorene, as a HTL) was purchased from Lumtec.

### Synthesis of SZO nanocomposites

Pure ZnO, as a pristine sample, as well as tin–zinc-oxide (SZO) nanocomposites were synthesized by a co-precipitation method with the same recipe as mentioned in our previous published work.^30^ The recipe includes the adjustment of the pH to 11 for the solution mixture of zinc sulfate (ZnSO_4_) and ammonium hydrogen carbonate (NH_4_HCO_3_) solution (2 M) for ZnO materials using diethyl amine as a stabilizer. Then, different amounts of SnCl_2_ were added to obtain SZO materials with different molar ratios (0.05, 0.1 and 0.2%, w/w). After that, the obtained sediment (either ZnO or SZO) was filtered, cleaned many times with deionized water, and then dried at 60 °C for 24 h. Ultimately, the desiccated precursors of the as-prepared SZO nanopowders at different molar ratios of Sn^2+^ ions (0.05, 0.1, and 0.2%) were annealed at 500 °C for 1 h. The obtained powders were used to fabricate the desired photoanodes for solar cells after purification.

### Fabrication of electrodes and devices

All ZnO and SZO based PVSC devices were fabricated on FTO substrates (TEC7, Hartford, USA), which were chemically etched to obtain the desired pattern followed by ultrasonic cleaning for 30 min with detergent, acetone, and isopropyl alcohol (IPA, 99.9% Acros) and finally washed with deionized water for cleaning. The substrates were exposed to an ozone–UV lamp for 18 min to remove any organic leftover. After that, a layer of dense TiO_2_ was deposited on FTO substrates *via* spray pyrolysis to form a blocking layer. Then, films of bare ZnO or SZO (with different molar ratio contents of Sn: 0.05, 0.1, and 0.2%) as ETL photoanodes were prepared by dispersion of the obtained powders in absolute ethanol (1 : 8 w/v) and then filtered with a PVDF hydrophobic 0.45 mm filter and spin-coated at 5000 rpm for 60 s. To increase the hydrophilicity of the substrates, they were exposed again to an ozone–UV lamp for 18 min. Subsequently, the perovskite films were prepared using one-step synthesis with the same sequence and steps according to the literature.^35^ As a hole transport layer (HTL), spiro-OMeTAD solution, doped with Li-TFSI in acetonitrile and 4-*tert*-butylpyridine, was dissolved in chlorobenzene, and then deposited *via* spin coating at 2000 rpm for 30 s.^35^ The final step was to evaporate aluminum (thickness ∼150 nm) on the top of the devices through a shadow mask inside a thermal evaporation machine to complete the fabrication process; the active area of the (Al) electrodes in the fabricated device was 0.09 cm^2^ as shown in the schematic illustration in Fig. S1, in the ESI.†

### Structural and morphological characterization

XRD (Bruker axis D8 diffractometer) using Cu-Kα (*λ* = 1.5406) was used to elucidate the crystallinity and crystal structure of the synthesized materials. A FESEM (JEOL JSM-5410) and an AFM (Nanosurf C300 Controller Flex AFM, Switzerland) were used to identify the microstructure and surface roughness of the synthesized materials as well as for the cross-sectional imaging of the assembled solar cells, besides the EDX measurements of the obtained samples. The specific surface area (*S*_BET_), pore size, pore volume and average pore size were realized on an ASAP 2020 (Micromeritics Instruments, USA) nitrogen adsorption apparatus. X-ray photoelectron spectroscopy (XPS) studies were pursued by using a Thermo Scientific K-ALPHA, XPS machine, England to identify the chemical composition of the prepared materials. The Fourier transform infrared (FTIR) absorption spectra were acquired using a JASCO 3600 spectrophotometer.

### Optical and photovoltaic measurements

A UV vis-NIR scanning spectrophotometer (Jasco-V-570 Spectrophotometer, Japan) was used to measure the UV-vis absorption spectra of ETL materials as well as perovskites on different ETLs through a double beam machine using a reflectance accessory *via* filling the sample position with the desired sample deposited on an FTO substrate and the blank position filled with bare FTO. Moreover, PL spectra were monitored *via* a fluorescence spectrophotometer (Shimadzu RF-5301 PC, Japan). The PL measurements were performed with the help of an integrating sphere that created a light source with apparent uniform intensity over all positions of the measured samples. The *J*–*V* measurements were recorded using a solar simulator.^36^ External quantum efficiency (EQE/IPCE) was measured using a measurement system (PVE 300, Bentham). Moreover, electrochemical impedance spectroscopy measurements (EIS) were pursued with a computer-controlled potentiostat (EG&G, M273) equipped with a frequency response analyzer (EG&G, M1025).

### Conductivity measurements

An electrochemical analyzer (Potentiostat Model Parastat Princeton 4000) was used to detect the resistivity of the prepared materials. Resistivity was measured at several positions of the ETL surface from one direction and at the FTO part from the other direction. In order to get a precise comparison, a fixed distance between the ETL and FTO positions was applied. The reported results are the mean of almost 15 positions tested for each sample.

## Results and discussion

**Fig. 1:**
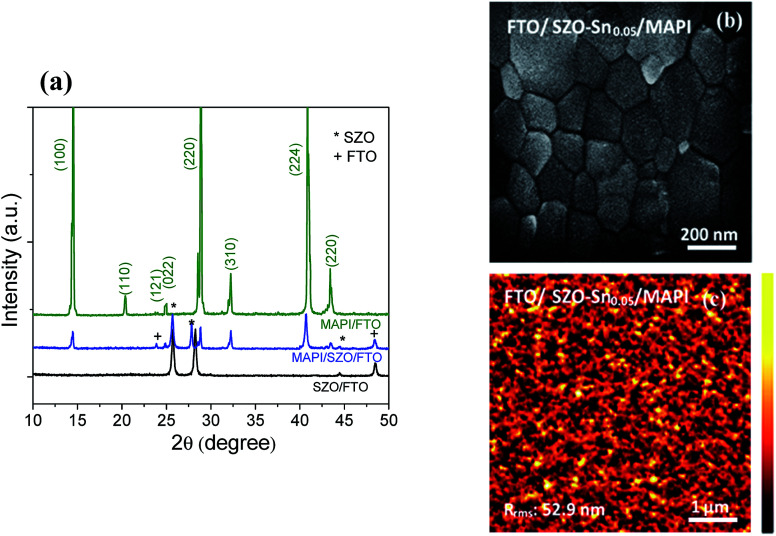
(a) XRD of SZO–Sn_0.05_ on FTO (black line), FTO/SZO–Sn_0.05_/CH_3_NH_3_PbI_3_ (blue line), and CH_3_NH_3_PbI_3_ on FTO (green line). The asterisks and the crosses refer to the planes of the SZO composite and FTO, respectively, (b) FESEM top-view image, and (c) AFM image of FTO/SZO–Sn_0.05_/MAPI.

To investigate the crystal structure of the synthesized SZO nanocomposites and the perovskite materials used in this study, their XRD spectra were recorded as depicted in Fig. 1a. The acquired results exhibit several diffraction peaks for the prepared materials, which are well matched with the diffraction peaks of the SnO–ZnO nanocomposite structure JCPDS (89-1397).^37^ No diffraction peaks related to SnO were detected indicating that the Sn^2+^ ions have substituted Zn^2+^ ions in the samples without changing its crystal structure. In addition, for CH_3_NH_3_PbI_3_ (MAPI) on SZO, the peaks marked with asterisks refer to the existence of the SZO nanocomposite in the tetragonal phase of the perovskite materials, which plainly appeared through the peaks at different 2*θ* positions with no impurities.^42^Fig. 1b shows the surface morphology of the spin-coated perovskite film on SZO–Sn_0.05_ with grain sizes ranging from 500 nm to 800 nm, indicating the good crystallinity of the perovskite materials.^36^ Moreover, the AFM imaging of the perovskite film on SZO confirms the homogeneity and the large grain boundaries of the obtained film with a root-mean-square roughness (*R*_rms_) of 52.9 nm as shown in Fig. 1c.

**Fig. 2:**
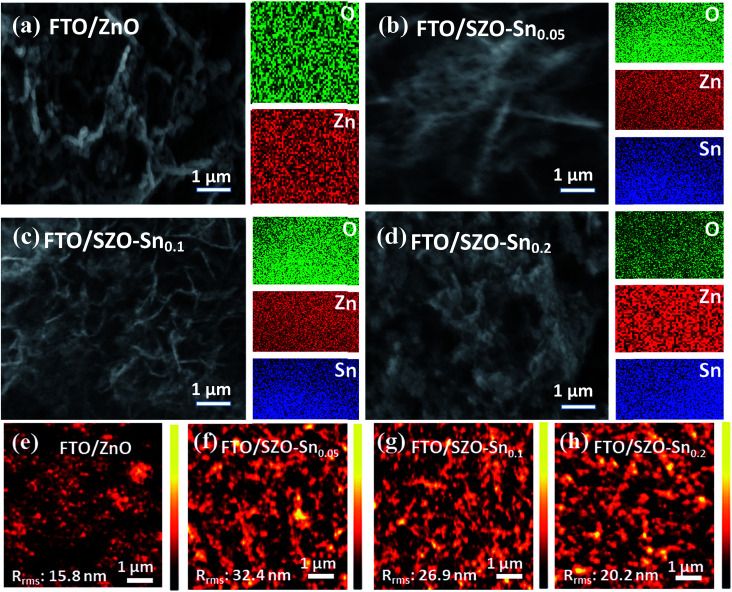
FESEM and EDX mapping of (a) pristine ZnO, (b) SZO–Sn_0.05_, (c) SZO–Sn_0.1_, and (d) SZO–Sn_0.2_. (e–h) AFM of the ZnO, SZO–Sn_0.05_, SZO–Sn_0.1_ and SZO–Sn_0.2_ ETLs, on FTO substrates respectively.

Fig. 2a–d show the FESEM and elemental mapping images of bare ZnO and SZO layers with different Sn : Zn molar ratio compositions. The images reveal the formation of spherical nanoparticles with sizes in the range of 15–30 nm which are agglomerated in the form of a panicle-like morphology. While substituting Zn with Sn resulted in no change in the overall morphology, the particle size of the nanocomposites was slightly decreased. This decrease in the particle size should increase the surface area of the nanocomposites, which is favorable to enhance the efficiency of the assembled solar cells using these materials. Furthermore, the Sn content in the nanocomposites and its distribution are investigated using EDX mapping. The EDX results in the case of ZnO nanoparticles, Fig. 2a, indicate the presence of Zn and O elements and their homogeneous distribution. Moreover, the elemental mapping of SZO–Sn_0.05_, SZO–Sn_0.1_, and SZO–Sn_0.2_, respectively, demonstrates that the weight percent of O (green) varied from = 15.1 to 26.1 wt%, Zn (red) varied from 84.8 to 61.4 wt%, and Sn (blue) varied from 0 to 20.6 wt%. These results confirm the uniform distribution of Sn in the ZnO. The atomic and weight percentages of the elemental composition of the fabricated nanocomposites are listed in Table S1, in the ESI. Fig. 2e–h show the corresponding AFM images, confirming the homogeneity of the fabricated films with different root-mean-square roughness (*R*_rms_) values that depend on the Sn content in the film, being 15.8 nm, 32.4, 26.9, and 20.2 nm for pure ZnO, SZO–Sn_0.05_, SZO–Sn_0.1_, and SZO–Sn_0.2_, respectively. The surface roughness of each SZO film was thus slightly more rugged than that of the pristine ZnO counterpart and increased with decreasing the Sn content, showing the highest value for SZO–Sn_0.05_ samples. After deposition of different ETLs on the FTO substrate, nucleation of the crystals occurred and the grains replicated, which may cause the increase in the film roughness upon changing the Sn content in the SZO material structure.^30,37–39^

**Fig. 3:**
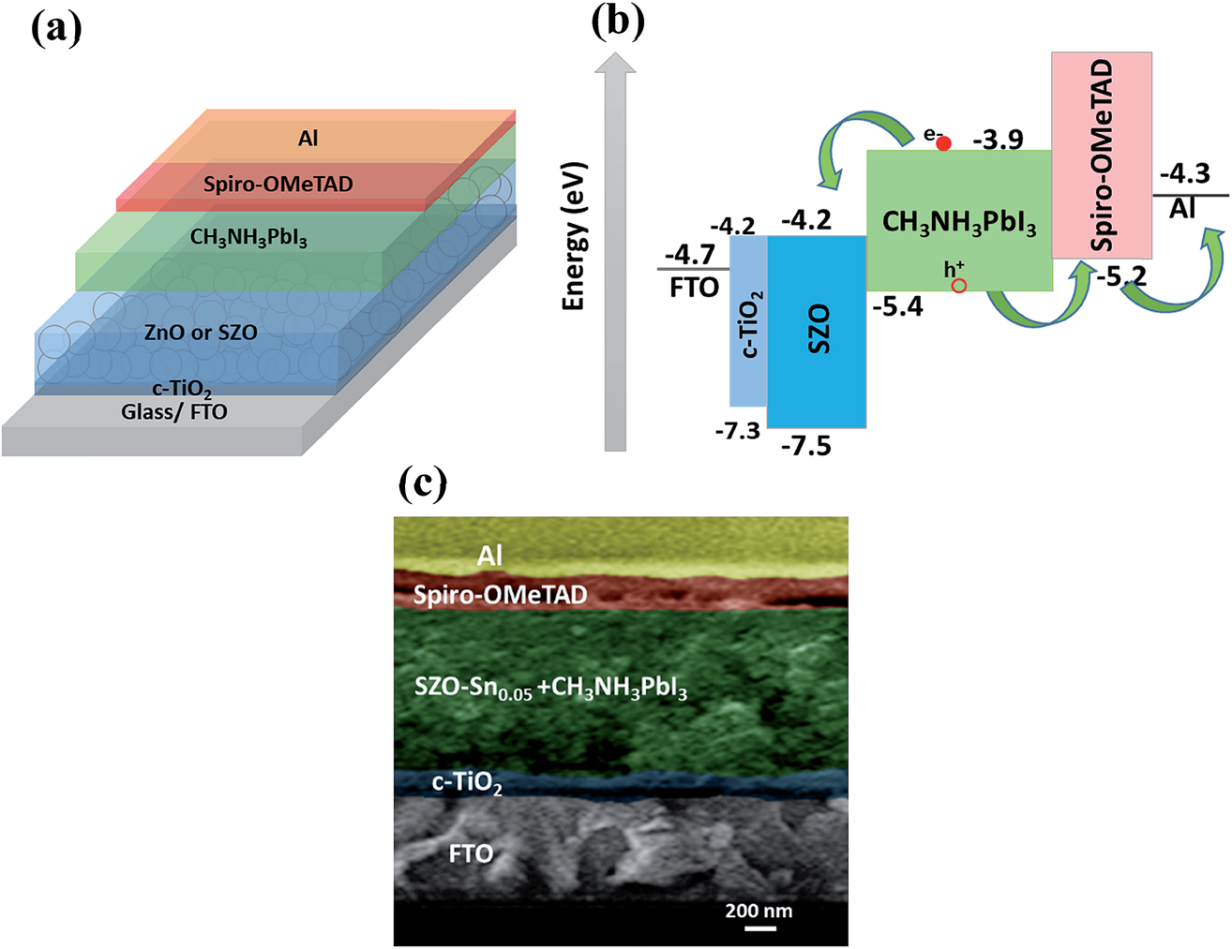
(a) Schematic diagram, (b) energy level diagram, and (c) cross-sectional FESEM of the assembled solar cells with the configuration of FTO/c-TiO_2_/SZO–Sn_0.05_/CH_3_NH_3_PbI_3_/spiro-OMeTAD/Al.

**Fig. 4:**
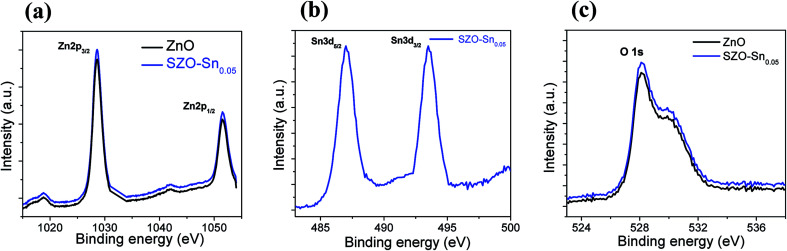
Narrow-range X-ray photoelectron spectra (XPS) of (a) Zn 2p_3/2_ and Zn 2p_1/2_ for ZnO and SZO–Sn_0.05_ samples; (b) Sn 3d_5/2_ and Sn 3d_3/2_ for the SZO–Sn_0.05_ sample; and (c) O 1s for ZnO and SZO–Sn_0.05_ samples.

Fig. 3a shows a schematic representation of the assembled perovskite solar cell with a device layout of FTO/c-TiO_2_/ETL/CH_3_NH_3_PbI_3_ (MAPI)/spiro-OMeTAD/Al, and ETL is ZnO or SZO. The corresponding energy level diagram of the perovskite solar cell components depending on ZnO or SZO as the ETL is illustrated in Fig. 3b. The reported conduction band energy of ZnO is 4.2 eV and SnO_2_ is 4.3 eV, which is very close to that of SZO (4.2 eV).^30^ Note that these values are lower than the value of the lowest occupied molecular orbital (LUMO) of CH_3_NH_3_PbI_3_ (3.9 eV), and thus electrons in the perovskite absorber material can easily transfer to the ETL, ensuring efficient dissociation of free charge carriers at the ETL/CH_3_NH_3_PbI_3_ interface.^31,33^Fig. 3c shows a cross-sectional FESEM image of the assembled solar cell with the configuration of FTO/c-TiO_2_/SZO–Sn_0.05_/CH_3_NH_3_PbI_3_/spiro-OMeTAD/Al. The homogeneity of each layer and the good contact between them pointed out that the charge separation and movement in the device will occur easily, which should enhance the overall efficiency of the assembled devices. Furthermore, XPS measurements were performed to investigate the composition of the deposited SZO on FTO and perovskite on SZO films, Fig. 4a–c and S2a and b in the ESI.† The results show the narrow spectral peaks of Pb 4f and I 3d for MAPI,^35^ Fig. S2a and b,† as well as the O 1 s, Zn 2p, and Sn 3d peaks for SZO, Fig. 4a–c.^30^ These peaks confirm the existence of all expected components in the deposited materials. The Zn 2p_3/2_ 2p_1/3_ peaks either for ZnO or SZO–Sn_0.05_, Fig. 4a, at approximately 1029.6 and 1050.3 eV, respectively, are characteristic of Zn–O bonds,^30^ which confirms the existence of ZnO composed with SnO nanoparticles by a facile solution processable wet chemical method. In addition, the XPS spectra of Sn3d for SZO–Sn_0.05_ (Fig. 4b) indicated the presence of spin orbit components 3d_3/2_ and 3d_5/2_ at binding energies of 486.86 and 493.56 eV, respectively.^30^ Moreover, Fig. 4c shows the O 1s spectra of ZnO and SZO–Sn_0.05_ samples. The incorporation of Sn^2+^ ions into the SZO structure can reinforce the contact barrier between the perovskite and SZO layers due to its unique chemical activity,^35^ which facilitates the electron transfer and thus enhances the efficiency of the fabricated devices.

Moreover, N_2_ sorption BET measurements were used to assess the mesoporous structure of the obtained nanocomposites *via* the huge number of small pores that exist between the nanoparticles, resulting in a higher surface area than that of pristine ZnO. The nitrogen sorption–desorption isotherm analysis of our materials shows specific surface areas (*S*_BET_) of 34.04, 49.12, 61.37, and 65.16 m^2^ g^−1^, pore volumes of 0.36, 0.35, 0.41, and 0.39 cm^3^ g^−1^ and pore sizes of 42.3, 29.3, 26.8, and 24.1 nm for pure ZnO, SZO–Sn_0.05_, SZO–Sn_0.1_, and SZO–Sn_0.2_, respectively. The obtained results indicate a direct proportion between the pore size, the pore volume, and the specific surface area.

Fourier-transform infrared (FTIR) spectroscopy was employed to further characterize the fabricated SZO–Sn_0.05_ film as an example of SZO nanocomposite materials, Fig. S3, ESI.† While the peaks observed in the range 1000 and 1500 cm^−1^ can be ascribed to the vibration mode of Sn–O, the sharp and strong band appearing around 669 cm^−1^ is characteristic of the stretching mode of Zn–O. In addition, the presence of –OH groups appears plainly *via* the obtained peaks corresponding to the stretching vibration of O–H and bending vibrations of adsorbed water molecules at around 3300–3450 cm^−1^ and at 1640 cm^−1^, respectively.^40^ Furthermore, impurities like organic residues and contaminants, –CH and –CH_2_, are not observed in the obtained spectra, indicating the high purity of the synthesized materials.

**Fig. 5:**
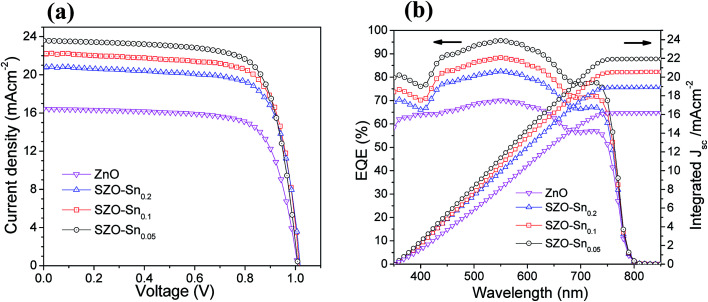
(a) *J*–*V* curves, (b) EQE spectra and integrated current density of pristine ZnO, SZO–Sn_0.05_, SZO–Sn_0.1_ and SZO–Sn_0.2_, respectively, as effective ETLs for the best PVSCs.

The effect of Sn-substitution on the photovoltaic performance of the assembled devices is examined under simulated AM1.5 G illumination (100 mW cm^−2^) as shown in (Fig. 5). The current–voltage (*J*–*V*) and IPCE with integrated *J*_sc_ measurements of the solar cells with ZnO or SZO (0.05, 0.1, and 0.2 molar ratio contents of Sn, *i.e.* pure ZnO, SZO–Sn_0.05_, SZO–Sn_0.1_, and SZO–Sn_0.2_, respectively) as an ETL are compared to each other and the relevant photovoltaic parameters are summarized in Table 1. The SZO-based PVSCs showed better performance compared to that based on bare ZnO. The power conversion efficiency (PCE) of all the tested devices was found to be increased with increasing the Sn content in the ETL. Note that the PCE was mainly dependent on the enhancement in *J*_sc_ upon the incorporation of Sn, which may be ascribed to (1) the positive shift in the Fermi level upon Sn incorporation, which improved the electron injection efficiency and reduced the interfacial resistance and (2) the larger surface area of SZO, resulting in better loading of the absorber materials with a better layer connection strength.^30,37,38^ Among all devices, the device containing the SZO–Sn_0.05_ ETL exhibits the highest efficiency of 17.81 with a short circuit current density (*J*_sc_) of 23.59 mA cm^−2^, an open-circuit voltage *V*_oc_ of 1 V, and a fill factor (FF) of 0.754. In addition, by measuring the forward scan to check the hysteresis behavior of our SZO solar cells, a small effect of hysteresis was noticed for these SZO devices, especially for SZO–Sn_0.05_-based devices compared to the ZnO-based devices, Fig. S4, ESI.† The scan range with varied initial bias plays an important role in the hysteretic behavior and can alter the PV performance.^35^ The small difference in *J*_sc_ between the reverse and forward scans is one possible reason behind the low hysteresis effect observed, Fig. S4, ESI,† which originates from the low applied voltage to these cells. Upon increasing the Sn content to 10 and 20%, *i.e.* SZO–Sn_0.1_ and SZO–Sn_0.2_, respectively, the cell performance deteriorates, which can be attributed to the formation of a separate SnO phase that could act as a charge trapping site for electron−hole recombination.^30,37^ On the other hand, the IPCE measurements also support the dramatic improvement in the photocurrent density (Fig. 5b). The incorporation of the SZO–Sn_0.05_ ETL in the device structure resulted in more than 90% improvement in the IPCE in the wavelength range of 400–800 nm, whereas the photocurrent was dramatically reduced when the Sn content exceeds 5%, *i.e.* SZO–Sn_0.1_ and SZO–Sn_0.2_. The reduction in photocurrent at a higher Sn content is mainly related to the reduction in the perovskite loading and inefficient electron injection from the perovskite to the ETL.^39^Fig. 5b shows an integrated *J*_sc_ of 16.17, 21.94, 20.56, and 18.90 mA cm^−2^ for the devices made of bare ZnO, SZO–Sn_0.05_, SZO–Sn_0.1_ and SZO–Sn_0.2_ ETLs, respectively. These *J*_sc_ values are in agreement with the experimentally obtained *J*_sc_ for the assembled cells within the experimental uncertainties, which indicate the plausible method of fabrication.

**Table 1:** Photovoltaic parameters of *J*_sc_, *V*_oc_, FF, and PCE of PVSCs based on ZnO, SZO–Sn_0.05_, SZO–Sn_0.1_ and SZO–Sn_0.2_ materials as ETLs

Devices^a^	*J*_sc_/mA cm^−2^	*V*_oc_/V	FF	PCE%^b^	*E*_g_ (eV)
ZnO	17.43	0.99	0.696	12.02 (11.73 ± 0.25)	3.15
SZO–Sn_0.05_	23.59	1.00	0.754	17.81 (17.21 ± 0.42)	3.05
SZO–Sn_0.1_	22.18	1.00	0.715	15.86 (15.44 ± 0.36)	3.10
SZO–Sn_0.2_	20.78	0.98	0.677	13.80 (13.20 ± 0.36)	3.10

a The devices were fabricated using ZnO, SZO–Sn_0.05_, SZO–Sn_0.1_ and SZO–Sn_0.2_ materials as electron transfer layers (ETLs).

b The average values shown in parentheses were obtained from 30 devices fabricated under the same experimental conditions for each ETL material.

**Fig. 6:**
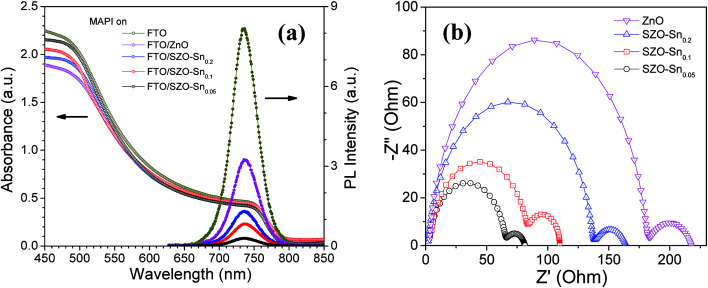
(a) Absorption spectrum and steady-state photoluminescence spectrum (PL) of the perovskite (MAPI) on FTO, bare ZnO and SZO–Sn_0.05_, SZO–Sn_0.1_ and SZO–Sn_0.2_ films, respectively; (b) Nyquist curve impedance spectra of the best PVSC devices based on pristine ZnO and SZO–Sn_0.05_, SZO–Sn_0.1_ and SZO–Sn_0.2_ films, respectively.

To better understand the difference in the photovoltaic performance of the tested devices, absorption and steady-state photoluminescence (PL) measurements were performed as convenient tools to investigate the charge carrier trapping, migration and transfer.^41^Fig. 6a demonstrates the PL and absorption spectra of the perovskite absorber layer [CH_3_NH_3_PbI_3_, (MAPI)] deposited on the bare FTO glass, ZnO or SZO ETLs. The perovskite films with the SZO–Sn_0.05_ ETL show the highest absorption values compared with the other films, probably due to the higher surface area of the SZO mesoporous structure that enables better light harvesting. Note that the emission peaks appearing at ∼740 nm originate from MAPI. The quenching in the PL intensity, compared to bare FTO, is due to the contact between CH_3_NH_3_PbI_3_ and the ETL layer, indicating good charge extraction across the interface.^42^ The more significant decrease in the intensity for SZO samples compared to the bare ZnO counterpart indicates more efficient electron transfer from the perovskite layer to SZO, proving that Sn substitution can successfully enhance the electron extraction rate at the ETL/MAPI interface.^43,44^ The results demonstrate that SZO–Sn_0.05_ exhibits the strongest PL quenching efficiency, the best injection ability, and the lowest recombination behavior, which would help to enhance the overall performance of the assembled perovskite solar cells. Furthermore, the results demonstrate that SZO–Sn_0.05_ exhibits the strongest PL quenching efficiency, the best injection ability, and the lowest recombination behavior, which would help to enhance the overall performance of the assembled perovskite solar cells. As the material absorbs more light, the number of photogenerated charge carriers increases. If those charge carriers are well separated with minimum recombination, the probability of PL quenching increases. This result, in conjunction with the SZO–Sn_0.05_ sample having the highest absorption of the various devices, gives the best overall performance of the SZO–Sn_0.05_ sample. In addition, the optical transmittance and the band-gap energy (*E*_g_) of the different ETLs and MAPI can be evaluated from the Tauc plots shown in Fig. S5, ESI.† The band gap is red-shifted from 3.15 to 3.05 eV for ZnO and different SZO films as illustrated in Table 1. This decrease in the band gap results in an enhancement in the photoconversion efficiency of the SZO films due to the easy movement of electrons and holes between materials in the device structure.^45,46^

To further assess the charge separation and charge recombination in the prepared ETL materials, electrochemical impedance spectroscopy (EIS) measurements were performed as shown in Fig. 6b. The obtained results show two semicircles, one in the high frequency range representing the carrier-transport resistance (*R*_1_) at the interface between the perovskite layer and the ETL when the same hole transport material (spiro-OMeTAD) was used, and the other one is related to the charge recombination resistance (*R*_2_) at the SZO/MAPI interface.^47,48^ While *R*_1_ is lowest for the device made using the SZO–Sn_0.05_ composite film, the device with a high content of Sn (SZO–Sn_0.2_) exhibits a larger *R*_1_, because a high content of Sn deteriorates the conductivity of the ZnO film as shown in Fig. 6b. Upon increasing the concentration of Sn, the recombination resistance increases gradually and the device with the SZO–Sn_0.05_ composite film has the smallest *R*_2_, which probably occurs because the low concentration of Sn impurities reduces the recombination of the charge carriers.

Note that the obtained PCE results (Table 1) are in good correlation with the roughness of the ETLs. Moreover, previous studies claimed that if the conductivity of the ETL increases, the net efficiency of the assembled devices would increase.^49^ Therefore, to emphasize the relationship between roughness and conductivity and their impact on the device performances, the resistivity of ZnO and SZO was measured using an electrochemical analyzer (Potentiostat Model Parastat Princeton 4000). The resistivities of ZnO, SZO–Sn_0.05_, SZO–Sn_0.1_ and SZO–Sn_0.2_ were found to be 90, 24, 37, and 62 Ω cm^−1^, corresponding to conductivities of 11.11, 41.66, 27.02, and 16.12 mS m^−1^, respectively. In addition, Fig. S6, ESI† illustrates the correlation between the PCE and the roughness of the different ETLs and their conductivity. Note the good interconnection between the roughness and conductivity of the different ETLs and the obtained PCEs of the assembled cells, where increasing the roughness and conductivity of the ETL resulted in the increase of the net PCE of the devices.

**Fig. 7:**
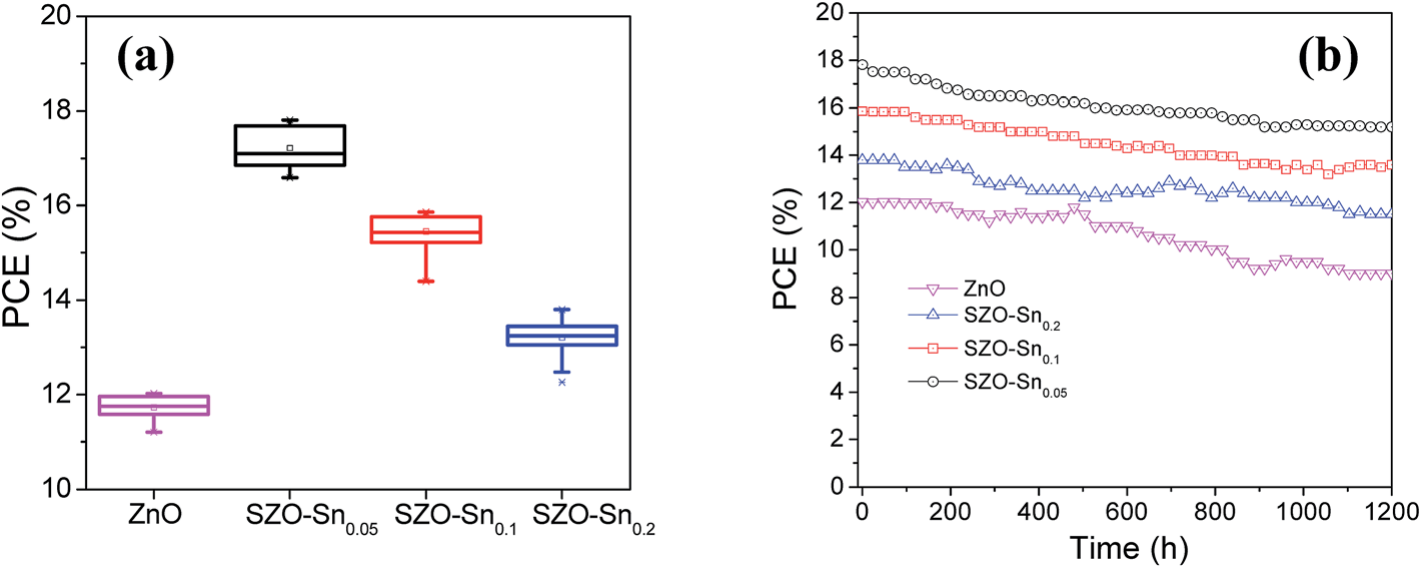
(a) Box chart of PCEs for the 30 solar cell devices and (b) efficiency of encapsulated devices as a function of the storage time.

Fig. 7a and S7a–c, ESI† show the average photovoltaic parameter distribution histogram diagrams of 30 devices, demonstrating very good reproducibility with limited variation. The obtained average PCEs with the corresponding standard deviation are 11.73 ± 0.25, 17.21 ± 0.42, 15.44 ± 0.36, and 13.20 ± 0.36% for the devices using bare ZnO, SZO–Sn_0.05_, SZO–Sn_0.1_ and SZO–Sn_0.2_ as ETLs, respectively. The corresponding average *J*_sc_ values are 17.19 ± 0.53, 22.85 ± 0.75, 21.51 ± 0.68, and 19.95 ± 0.36 mAcm^−2^ (Fig. S7a, ESI†), and average *V*_oc_ values of 0.98 ± 0.014, 0.99 ± 0.003, 1.00 ± 0.001, and 0.98 ± 0.013 V (Fig. S7b, ESI†) and average FF of 69.21 ± 3.03, 75.40 ± 2.08, 71.83 ± 1.56, and 67.09 ± 1.63 (Fig. S7c, ESI†) as listed in detail in Tables S2–S5, ESI.† The highest PCE was found for the device using SZO–Sn_0.05_ as an ETL, mainly due to the highest *J*_sc_, which is because of the most efficient interfacial charge transfer and the lowest carrier recombination as confirmed from the EIS measurements (Fig. 6b).^31,50^Fig. 7b shows the stability of the assembled solar cell devices made using bare ZnO or SZO ETLs during storage in ambient air environment at room temperature over ∼1200 h. Although the four devices showed almost similar stability in the first 600 h, increasing the storage time reduces the PCE of bare ZnO-devices more significantly than the SZO-device counterparts, where the efficiency decreased from 12.02 to 9%, accounting for 30% loss. However, the SZO-devices with 5% Sn retained ∼85% of their initial efficiency of 17.81% under the same conditions. Therefore, it is clear that the introduction of Sn into ZnO, which can act as a shield to prevent the dissolution of the perovskite layer, results in the best stability of PVSCs compared to other materials studied elsewhere.^31^

## Conclusion

We demonstrated the positive effect of Sn substitution into the ZnO structure for use as an efficient ETL for PVSCs. The fabricated cells using ETLs containing 5, 10 and 20% Sn, *i.e.* SZO–Sn_0.05_, SZO–Sn_0.1_, and SZO–Sn_0.2_, respectively, showed a relatively high photovoltaic performance with a maximum PCE of 17.81% compared to 12.02% for the PVSC based on bare ZnO as the ETL. Quenching of the photoluminescence in the Sn-containing devices suggested efficient extraction of the charge carriers with the suppression of the electron–hole recombination at the SZO/CH_3_NH_3_PbI_3_ interface. The obtained high PCE was further asserted *via* electrochemical impedance spectroscopy measurements, which indicated low recombination resistance in the devices. More importantly, 30 devices were thoroughly tested to investigate the reproducibility of the devices' performance, indicating that the SZO–Sn_0.05_ films exhibited the best durability as compared to bare ZnO. Finally, the presence of Sn in the ZnO lattice enhanced not only the photovoltaic performance but also the photo-stability of PVSCs as revealed by the long-term stability tests for 1200 h.

## Funding

This work was funded by the Science & Technology Development Fund in Egypt (STDF), grants no. 33338 and 25250.

## Conflicts of interest

There is no competing financial interest declared.

## Supplementary Material

NA-001-C9NA00182D-s001
