# Expression of concern: Tin–zinc-oxide nanocomposites (SZO) as promising electron transport layers for efficient and stable perovskite solar cells

**DOI:** 10.1039/d3na90114a

**Published:** 2023-11-27

**Authors:** Ahmed E. Shalan, Ayat N. El-Shazly, Mohamed M. Rashad, Nageh K. Allam

**Affiliations:** a Central Metallurgical Research and Development Institute (CMRDI) P. O. Box 87 11422 Helwan Cairo Egypt a.shalan@cmrdi.sci.eg; b Energy Materials Laboratory, School of Sciences and Engineering, The American University in Cairo (AUC) 11835 New Cairo Egypt nageh.allam@aucegypt.edu

## Abstract

Expression of concern for ‘Tin–zinc-oxide nanocomposites (SZO) as promising electron transport layers for efficient and stable perovskite solar cells’ by Ahmed E. Shalan *et al.*, *Nanoscale Adv.*, 2019, **1**, 2654–2662, DOI: https://doi.org/10.1039/C9NA00182D.

The Royal Society of Chemistry is publishing this Expression of concern in order to alert readers that concerns have been raised regarding the reliability of the FESEM data in Fig. 3c, XPS data in Fig. 4a and c, the EQE spectra in Fig. 5b, and the Nyquist curves in Fig. 6b. An investigation is underway, and an Expression of concern will continue to be associated with the article until a final outcome is reached.

Jeremy Allen

20^th^ November, 2023

Executive Editor, *Nanoscale Advances*.

## Supplementary Material

