# Supplementary material for: Tin–zinc-oxide nanocomposites (SZO) as promising electron transport layers for efficient and stable perovskite solar cells
Source: Nanoscale Adv. 2019 May 22;1(7):2654–62. doi: 10.1039/c9na00182d (PMC9418726; doi:10.1039/c9na00182d)
Supplement: NA-001-C9NA00182D-s001 [file NA-001-C9NA00182D-s001.pdf]

Electronic Supplementary Material (ESI) for Nanoscale Advances.
This journal is © The Royal Society of Chemistry 2019

Supporting Information

Tin-Zinc-Oxide Nanocomposites (SZO) as Promising Electron Transport Layers for Efficient and Stable Perovskite Solar Cells

Ahmed E. Shalan[1†], Ayat N. El-Shazly[1,2†], Mohamed M. Rashad[1] and Nageh K.Allam[2*]*

[1] Central Metallurgical Research and Development Institute, P.O. Box 87, 11422, Helwan, Cairo, Egypt.
[2] Energy Materials Laboratory, School of Sciences and Engineering, The American University in Cairo, 11835, New Cairo, Egypt.

[*] Corresponding Author: E-mail: (A. E. Shalan) a.shalan@cmrdi.sci.eg
E-mail: (N. K. Allam) nageh.allam@aucegypt.edu.
† (A. E. Shalan) and (A. N. El-Shazly) contributed equally to this work

Table of contents

Table S1 Summary of the weight and atomic percentage of the produced nanocomposites obtained from the EDX analysis……………………………..3

Figure S1: Schematic diagram of LbL of the assembled PVSCs………………………..4

Figure S2: XPS analysis of MAPI on FTO………………..…………………………5

Figure S3: FTIR spectra of SZO-$Sn_{0.05}$…………………….………………………...6

Figure S4: Hysteresis effect of J-V curve for different ETLs………………………….. 7

Figure S5: Optical transmittance and band gaps of the studied materials………………8

Figure S6: Correlation of the PCE, roughness, and conductivity of different HTLs …9

Figure S7: Box charts of 30 PVSCs assembled devices ………………………………10

Tables S2-S5: The calculated photovoltaic parameters…………………………….11-14

Table S1: Energy dispersive X-ray spectroscopy (EDX) analysis data for the produced materials of ZnO, SZO-$Sn_{0.05}$, SZO-$Sn_{0.1}$ and SZO-$Sn_{0.2}$

	Element	Weight%	Atomic%		Element	Weight%	Atomic%
**ZnO**	**O K**	15.16	42.19	**SZO-$Sn_{0.05}$**	**O K**	19.84	51.01
	**Zn K**	84.84	57.81		**Zn K**	75.05	47.22
					**Sn L**	5.11	1.77
	**Total**	100.00			**Total**	100.00	

	Element	Weight%	Atomic%		Element	Weight%	Atomic%
**SZO-$Sn_{0.1}$**	**O K**	26.15	60.74	**SZO-$Sn_{0.2}$**	**O K**	18.00	50.27
	**Zn K**	63.20	35.93		**Zn K**	61.41	41.98
	**Sn L**	10.56	3.33		**Sn L**	20.59	7.75
	**Total**	100.00			**Total**	100.00	

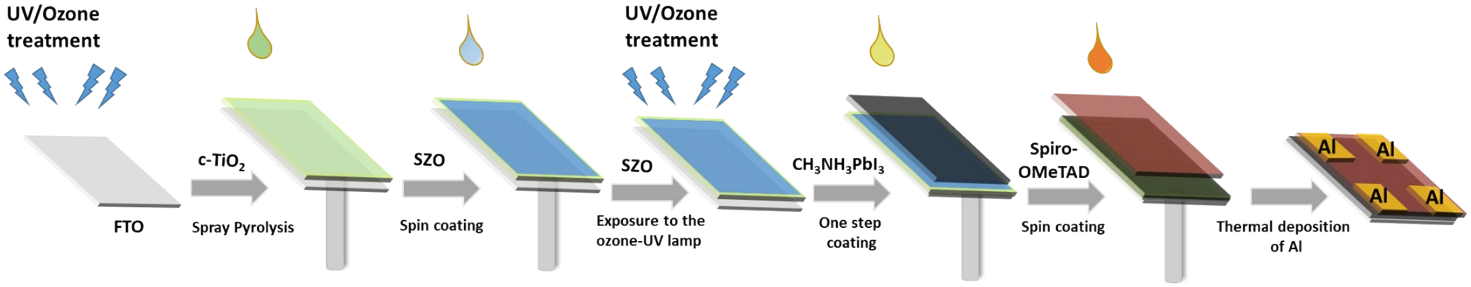

Figure S1: Schematic diagram of the layer-by-layer assembly of the PVSC with the configuration: glass/FTO/c-TiO_2/SZO//$CH_3NH_3PbI_3$/Spiro-OMeTAD/Al.

Figure S1 illustrate that, after cleaning the FTO substrate and deposition of the ETL (ZnO or SZO) over the blocking layer, the perovskite film was formed as a sensitizer layer for light absorption, heated to 70°C and kept for few minutes to evaporate the solvents and enhance the crystallinity of the perovskite film. The cell structure was completed by spin coating of organic HTL, followed by thermal evaporation of Al back electrode as discussed in details in the experimental section.

(a) **(b)**

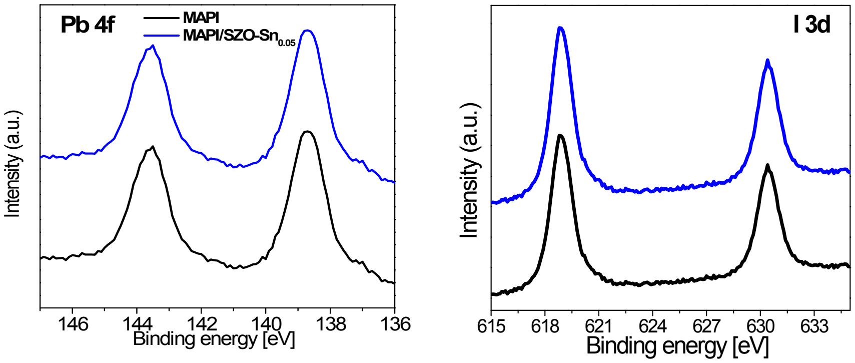

Figure S2: Narrow-range X-ray photoelectron spectra (XPS) of (a) Pb 4f and (e) I 3d for perovskite (MAPI) and MAPI on SZO-$Sn_{0.05}$.

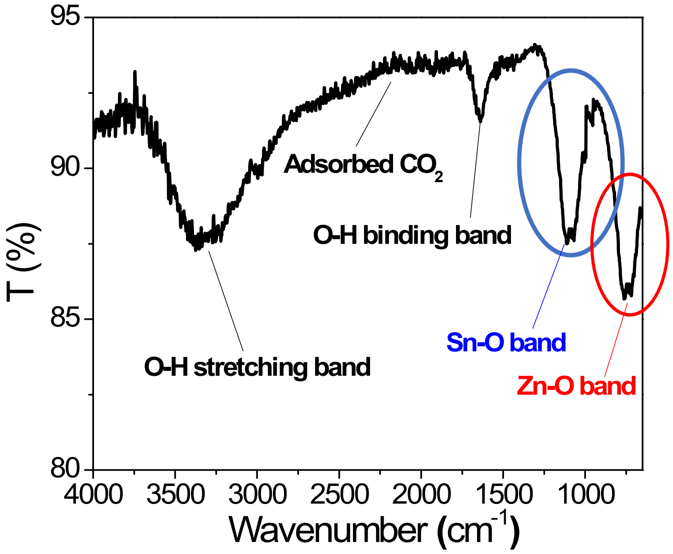

Figure S3: FTIR (T %) versus wavenumber (cm^{-1}) of SZO-$Sn_{0.05}$ nanocomposite.

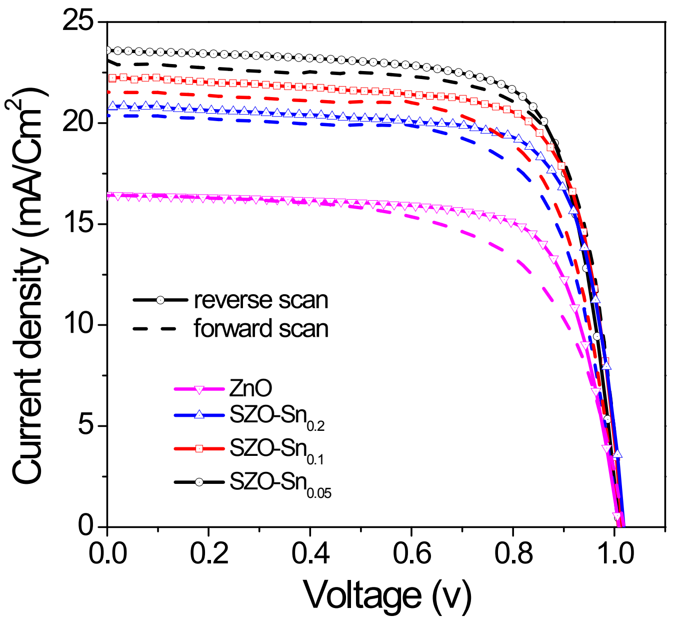

Figure S4: J-V curves (reverse and forward scan) showing the hysteresis effect of pristine ZnO, SZO-$Sn_{0.05}$, SZO-$Sn_{0.1}$ and SZO-$Sn_{0.2}$, respectively, as an effective ETLs for the best PVSCs.

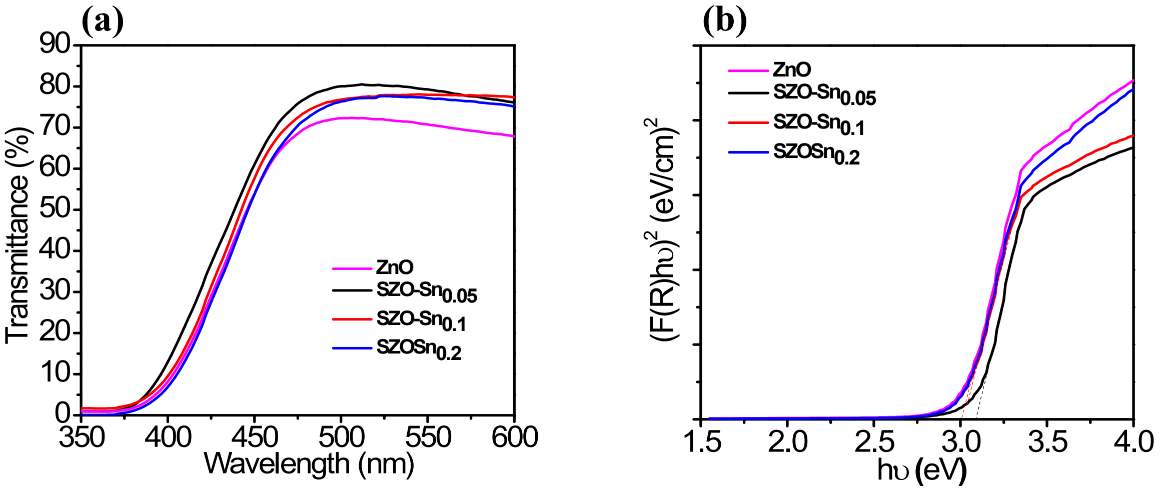

Figure S5: (a) Optical transmittance and (b) band gap of pristine ZnO, SZO-$Sn_{0.05}$, SZO-$Sn_{0.1}$ and SZO-$Sn_{0.2}$, respectively.

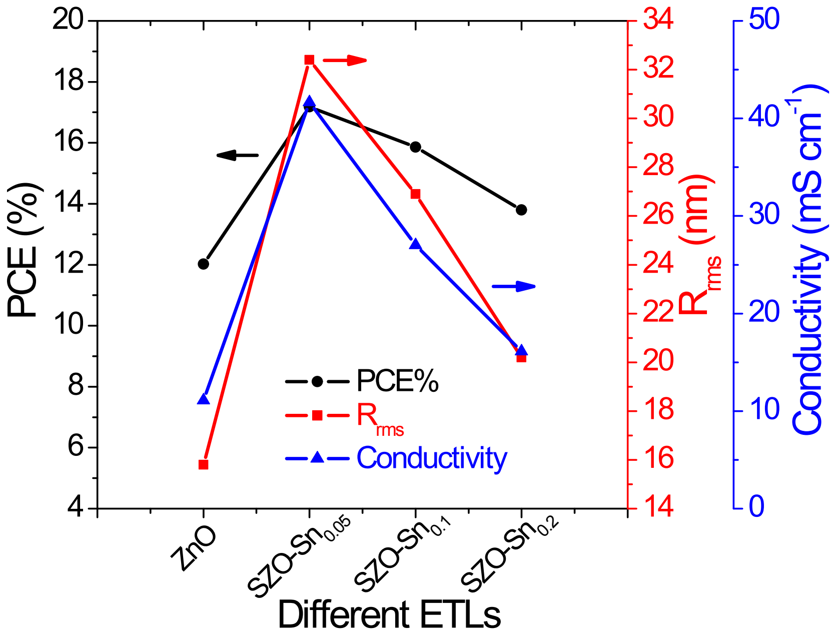

Figure S6. Correlation of device performance (PCE/%) (black line) with the different hole transfer layers (HTLs) films used in this study with respect to conductivity/mS cm^{-1} (blue line) and route mean square roughness R_{rms}/nm (red color).

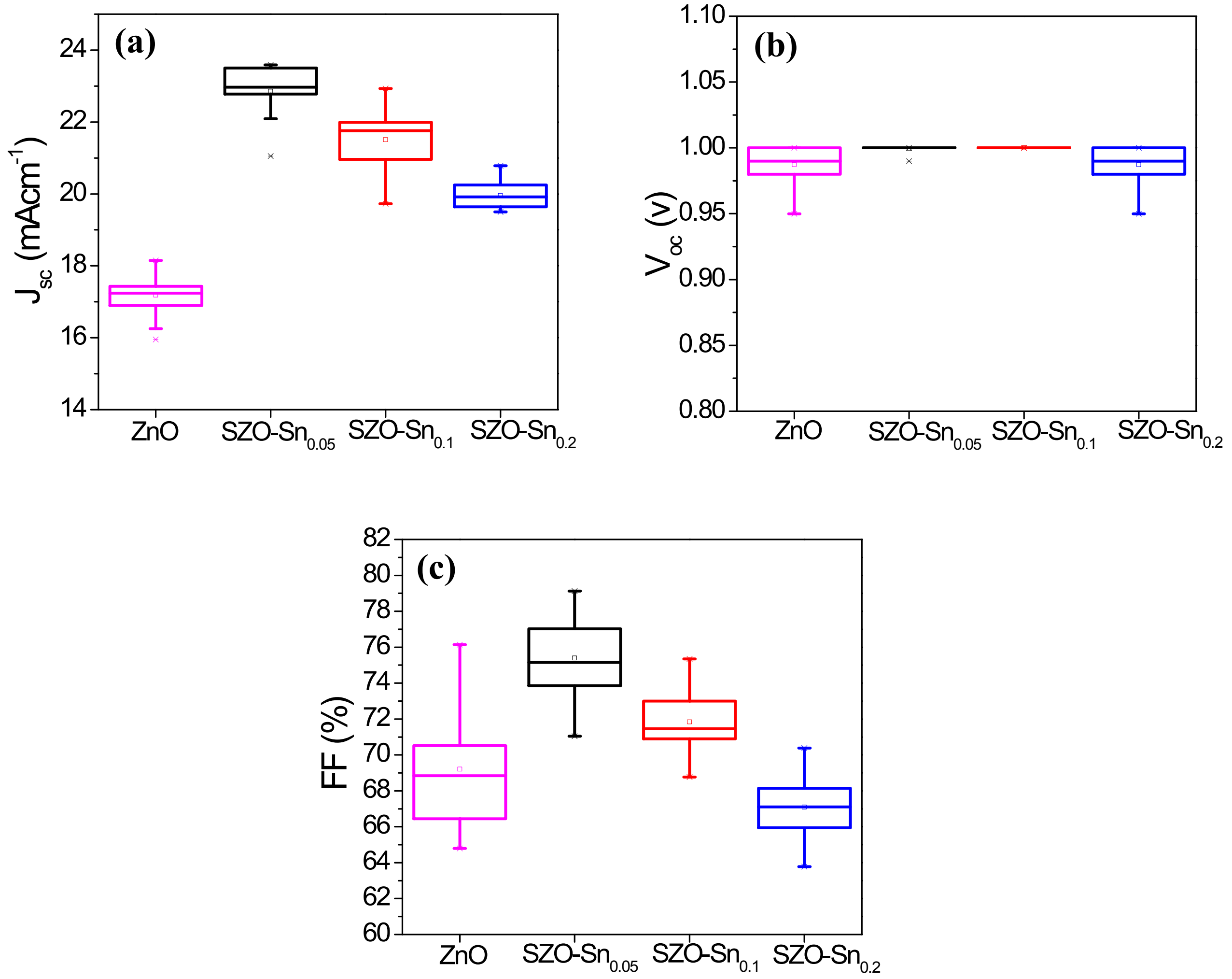

Figure S7. The J_{sc}, V_{oc} and FF box chats (a, b and c), respectively of the pristine ZnO (purple color), SZO-$Sn_{0.05}$ (black color), SZO-$Sn_{0.1}$ (red color) and SZO-$Sn_{0.2}$ (blue color), respectively.

Table S2. Photovoltaic parameters of thirty PVSCs devices made of pure ZnO under simulated AM-1.5G illumination (power density 100 mW cm^{-2}) with active area 0.09 cm^2.

# of cells	J_{sc}(mA/cm^2)	V_{oc} (V)	FF (%)	PCE (%)
1	17.43	0.99	69.66	12.02
2	17.24	0.99	70.19	11.98
3	17.39	0.99	69.41	11.95
4	17.42	0.98	69.58	12.00
5	17.43	1.00	68.84	12.00
6	17.28	1.00	69.15	11.95
7	16.69	0.95	75.43	11.96
8	16.34	0.95	74.08	11.50
9	15.95	0.96	76.14	11.66
10	16.56	0.99	71.18	11.67
11	16.68	0.97	72.94	11.80
12	16.25	0.99	73.78	11.87
13	17.25	0.99	70.32	12.01
14	17.39	1.00	67.74	11.78
15	18.10	1.00	66.13	11.97
16	18.15	1.00	64.79	11.76
17	17.65	1.00	65.60	11.58
18	17.82	0.99	66.20	11.68
19	17.87	0.99	67.54	11.95
20	17.90	0.98	66.81	11.72
21	16.90	0.98	70.52	11.68
22	17.42	0.99	65.81	11.35
23	17.43	0.98	68.78	11.75
24	17.10	0.98	68.62	11.50
25	16.90	0.99	71.72	12.00
26	17.12	1.00	65.42	11.20
27	16.95	1.00	68.96	11.69
28	16.80	1.00	68.45	11.50
29	16.99	1.00	65.92	11.20
30	17.16	0.99	66.45	11.29
**average**	17.19±0.53	0.98±0.014	69.21±3.03	11.73±0.25

Table S3. Photovoltaic parameters of thirty PVSCs devices made of SZO-$Sn_{0.05}$ under simulated AM-1.5G illumination (power density 100 mW cm^{-2}) with active area 0.09 cm^2.

**# of cells**	**J_{sc}(mA/cm^2)**	**V_{oc} (V)**	**FF (%)**	**PCE (%)**
1	23.59	1.00	75.49	17.81
2	23.50	1.00	73.61	17.30
3	23.54	1.00	73.27	17.25
4	22.90	1.00	74.23	17.00
5	23.01	1.00	74.31	17.10
6	22.95	1.00	72.28	16.59
7	22.66	1.00	74.66	16.92
8	21.37	1.00	78.38	16.75
9	23.24	1.00	72.63	16.88
10	23.55	1.00	74.90	17.64
11	22.89	1.00	76.40	17.49
12	23.55	1.00	71.04	16.73
13	23.00	1.00	73.86	16.99
14	22.87	1.00	73.41	16.79
15	22.78	1.00	73.96	16.85
16	22.97	1.00	77.49	17.80
17	23.45	1.00	75.69	17.75
18	23.46	1.00	75.66	17.75
19	21.38	1.00	77.92	16.66
20	22.09	1.00	79.13	16.69
21	21.05	1.00	75.55	16.88
22	21.55	1.00	77.91	16.79
23	21.55	1.00	78.84	16.99
24	23.50	1.00	75.53	17.75
25	23.33	0.99	77.02	17.79
26	23.00	1.00	76.95	17.70
27	22.80	0.99	78.37	17.69
28	23.55	1.00	74.73	17.60
29	22.90	1.00	75.15	17.21
30	23.55	1.00	73.63	17.34
**average**	22.85±0.75	0.99±0.003	75.40±2.08	17.21±0.42

Table S4. Photovoltaic parameters of thirty PVSCs devices made of SZO-$Sn_{0.1}$ under simulated AM-1.5G illumination (power density 100 mW cm^{-2}) with active area 0.09 cm^2.

# of cells	J_{sc}(mA/cm^2)	V_{oc} (V)	FF (%)	PCE (%)
1	22.18	1.00	71.46	15.86
2	22.00	1.00	71.81	15.80
3	21.99	1.00	72.07	15.85
4	21.90	1.00	71.23	15.60
5	21.15	1.00	70.63	14.94
6	21.15	1.00	73.04	15.45
7	22.10	1.00	68.77	15.20
8	21.98	1.00	69.42	15.26
9	21.84	1.00	71.84	15.69
10	21.65	1.00	70.66	15.30
11	22.93	1.00	68.90	15.80
12	21.90	1.00	72.42	15.86
13	21.00	1.00	71.38	14.99
14	21.10	1.00	70.99	14.98
15	22.15	1.00	70.83	15.69
16	22.18	1.00	71.23	15.80
17	22.18	1.00	71.00	15.75
18	20.90	1.00	75.35	15.75
19	21.95	1.00	71.38	15.67
20	20.96	1.00	73.47	15.40
21	21.76	1.00	70.90	15.43
22	21.84	1.00	70.32	15.36
23	21.90	1.00	71.96	15.76
24	21.12	1.00	74.81	15.80
25	20.89	1.00	73.00	15.25
26	19.73	1.00	72.93	14.39
27	20.70	1.00	73.47	15.21
28	20.80	1.00	72.83	15.15
29	20.96	1.00	73.23	15.35
30	20.36	1.00	73.82	15.03
**average**	21.51±0.68	1.00±0.001	71.83±1.56	15.44±0.36

Table S5. Photovoltaic parameters of thirty PVSCs devices made of SZO-$Sn_{0.2}$ under simulated AM-1.5G illumination (power density 100 mW cm^{-2}) with active area 0.09 cm^2.

# of cells	J_{sc}(mA/cm^2)	V_{oc} (V)	FF (%)	PCE (%)
1	20.78	0.98	67.76	13.80
2	19.70	0.99	68.96	13.45
3	20.72	0.98	65.99	13.40
4	19.60	0.99	68.03	13.20
5	19.66	0.98	66.69	12.85
6	19.55	0.98	68.16	13.06
7	20.05	0.98	67.43	13.25
8	19.98	0.99	65.21	12.69
9	20.20	0.99	63.90	12.78
10	19.64	0.99	68.09	13.24
11	20.29	0.99	65.66	13.19
12	19.78	1.00	66.93	13.24
13	19.64	1.00	65.88	12.94
14	19.99	1.00	68.43	13.68
15	20.34	0.99	68.13	13.72
16	19.92	0.98	70.38	13.74
17	20.25	0.97	69.69	13.69
18	19.50	0.95	66.18	12.26
19	19.61	0.95	66.99	12.48
20	19.63	0.99	68.79	13.37
21	19.94	0.98	68.93	13.47
22	19.70	0.99	67.63	13.19
23	19.75	1.00	68.15	13.46
24	19.57	1.00	67.85	13.28
25	20.46	1.00	63.78	13.05
26	20.46	1.00	65.00	13.30
27	20.31	0.99	65.94	13.26
28	19.99	0.99	66.39	13.14
29	19.52	1.00	67.11	13.10
30	20.05	1.00	64.83	13.00
**average**	19.95±0.36	0.98±0.013	67.09±1.63	13.20±0.36
